# Unraveling the contributions to the neuromelanin-MRI contrast

**DOI:** 10.1007/s00429-020-02153-z

**Published:** 2020-10-22

**Authors:** Nikos Priovoulos, Stan C. J. van Boxel, Heidi I. L. Jacobs, Benedikt A. Poser, Kamil Uludag, Frans R. J. Verhey, Dimo Ivanov

**Affiliations:** 1 School for Mental Health and Neuroscience, Alzheimer Center Limburg, Faculty of Health, Medicine and Life Science, Maastricht University, Maastricht, Netherlands; 2 Department of Cognitive Neuroscience, Faculty of Psychology and Neuroscience, Maastricht University, Maastricht, Netherlands; 3 Gordon Center for Medical Imaging, Department of Radiology, Massachusetts General Hospital, Harvard Medical School, Boston, MA USA; 4 Center for Neuroscience Imaging Research, Institute for Basic Science and Department of Biomedical Engineering, Sungkyunkwan University, Seobu-ro 2066, Jangan-gu, Suwon, Republic of Korea; 5 Techna Institute and Koerner Scientist in MR Imaging, University Health Network, 121-100 College Street, Toronto, M5G 1L5 Canada

**Keywords:** Neuromelanin, Neuromelanin-MRI, Locus coeruleus, Substantia nigra, Magnetization transfer

## Abstract

The Locus Coeruleus (LC) and the Substantia Nigra (SN) are small brainstem nuclei that change with aging and may be involved in the development of various neurodegenerative and psychiatric diseases. Magnetization Transfer (MT) MRI has been shown to facilitate LC and the SN visualization, and the observed contrast is assumed to be related to neuromelanin accumulation. Imaging these nuclei may have predictive value for the progression of various diseases, but interpretation of previous studies is hindered by the fact that the precise biological source of the contrast remains unclear, though several hypotheses have been put forward. To inform clinical studies on the possible biological interpretation of the LC- and SN contrast, we examined an agar-based phantom containing samples of natural Sepia melanin and synthetic Cys-Dopa-Melanin and compared this to the in vivo human LC and SN. *T*_1_ and *T*_2_* maps, MT spectra and relaxation times of the phantom, the LC and the SN were measured, and a two-pool MT model was fitted. Additionally, Bloch simulations and a transient MT experiment were conducted to confirm the findings. Overall, our results indicate that Neuromelanin-MRI contrast in the LC likely results from a lower macromolecular fraction, thus facilitating interpretation of results in clinical populations. We further demonstrate that in older individuals *T*_1_ lengthening occurs in the LC.

**Electronic supplementary material:**

The online version of this article (10.1007/s00429-020-02153-z) contains supplementary material, which is available to authorized users.

## Introduction

The Locus Coeruleus (LC) and the Substantia Nigra (SN) are small, neuromelanin (NM)-rich gray matter nuclei in the brainstem that show alterations in several neurodegenerative and psychiatric diseases, such as Parkinson’s and Alzheimer’s (AD) disease or depressive disorder (Braak et al. 2011; Busch et al. 1997; Liu et al. 2017; Marcyniuk et al. 1986; Sara 2009). Because of their critical modulating role in cognition and behavior, as well as being part of the pathophysiology of several neurodegenerative diseases, there is an increasing interest in imaging the LC and the SN with the so-called NM-MRI, and understanding the biological correlates of the MRI contrast observed (a search in Pubmed for NM-MRI provides 160 hits, as of 07–08–2020). Prior work has reported MRI signal differences in the LC between younger and older individuals and between clinically healthy older individuals and patients with AD, Parkinson’s disease, sleep disturbances or psychosis (Cassidy et al. 2019; Clewett et al. 2016; Ehrminger et al. 2016; Liu et al. 2018; Sasaki et al. 2006; Takahashi et al. 2015). This suggests that the contrast of the NM-MRI method is able to detect microstructural alterations related to underlying mechanisms of aging or disease. Given the clinical interest in this contrast, understanding its underlying biological correlates is crucial for the interpretation of these MRI alterations and to examine the potential for the development of a quantitative MRI-based in vivo LC and SN marker. So far, this was challenging given the small size of the structures (particularly for the LC which is maximally 15 mm long and 2–3 mm in diameter (German et al. 1988)) and their lack of contrast with the surrounding tissue using standard MRI approaches.

The LC and the SN are typically imaged as hyperintensities in Turbo Spin Echo (TSE) acquisitions with short echo time (TE) (Sasaki et al. 2006). We recently demonstrated that the TSE LC contrast relates to incidental Magnetization Transfer (MT) contrast and applied an efficient high-resolution MT-weighted sequence to successfully image the LC at 7 T (Nakane et al. 2008; Priovoulos et al. 2018, 2019). MT can be described as a magnetization exchange between the short *Τ*_2_, macromolecules-bound water protons and the freely moving intra-cellular and extra-cellular protons (Henkelman et al. 1993; Sled and Pike 2001). In a typical MT experiment, the application of an off-resonance pulse preferentially saturates the protons of the bound pool, which, due to their restricted motion range and the variable magnetic fields they experience from adjacent nuclei and ions, show a short *Τ*_2_ and a broad resonance bandwidth around the Larmor frequency. The bound pool in turn saturates the free water pool through exchange, resulting in reduced signal if one excites the free water pool during that period. The MT contrast in LC and SN implies that this exchange differs compared to adjacent tissue, due to variations in pool sizes or exchange and decay rates (Trujillo et al. 2019). Manipulating the saturation train in terms of amplitude, frequency offset and exchange time can provide insight into these characteristics (Ramani et al. 2002) to probe the contrast correlates of the signal.

**Fig. 1:**
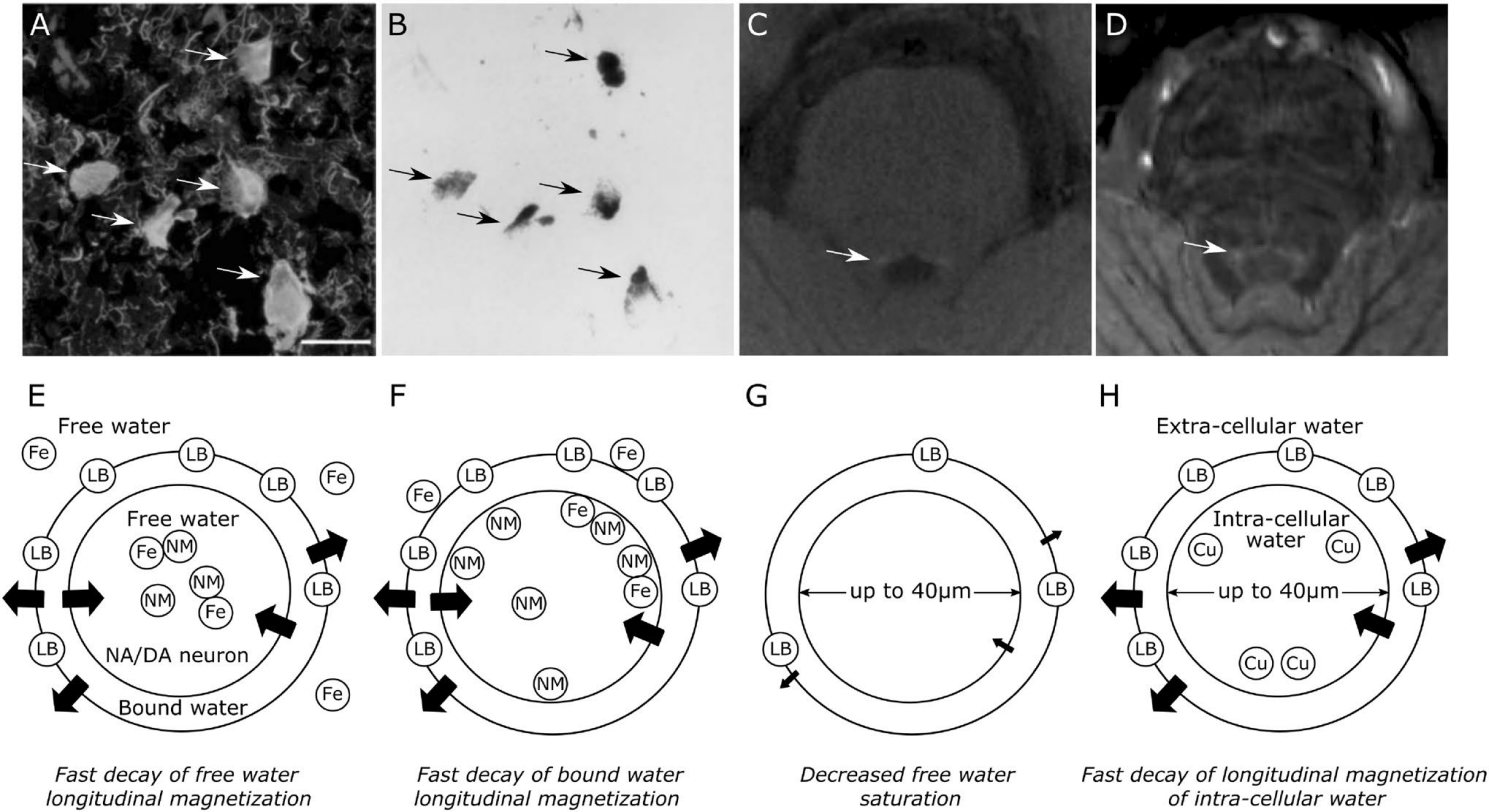
**a**–**b**, Photomicrography of human LC (modified with permission from (Kimberly Simpson, 2007)). **a** Tyrosine hydroxylase was used to visualize noradrenergic (NA) cells (white arrows). NA cells show a remarkably big diameter, up to 30–40 μm. **b** Melanin accumulation (black arrows) largely overlaps with the NA cells as shown in **a**. **c–d**, Axial slice of human brainstem at the level of LC (modified with permission from (Priovoulos et al. 2018)). **c** TSE with short TE is typically used to visualize the LC as hyperintense. **d** MT image of the same participant. A similar hyperintensity appears, indicating that the TSE is related to implicit MT. TSE/MT signal in LC and SN has been shown to spatially overlap with the NA (Keren et al. 2015) and dopaminergic (DA) neurons respectively (Kitao et al. 2013). **e**–**h**, Hypotheses for LC and SN MRI contrast following off-resonance saturation. The black arrows indicate the transfer of magnetization from the bound pool to the free water pool. **e** The presence of the NM and metals, for example iron bound to ferritin (Fe), may shorten the free water *T*_1_ so that the pool recovers faster from on- and off-resonance saturations and the LC and SN appear hyperintense. **f** The proximity of NM and NM-bound metals to the lipids of the membrane (LB) may shorten the *T*_1_ of the bound pool. **g** The LC and SN, as gray matter regions, may show a lower macromolecular content fraction due to less myelination (LB) and/or a large cellular body. **h** The unusually large NA and DA cells may result in a big pool of intra-cellular water protons. The presence of ions such as copper (Cu) within the NA neurons may shorten the *T*_1_ of the intra-cellular water pool causing the LC and SN to appear hyperintense

The adult LC and SN are unusual in that they are populated by large (up to 40 μm diameter; Fig. 1a) noradrenergic (NA) and dopaminergic (DA) neurons, which contain numerous organelles that can take up to 50% of the cell volume (Halliday et al. 2005) and are filled with NM macromolecules (Fig. 1b). NM is a large (~ 30 nm) polymer that is a by-product of noradrenaline (NE)/dopamine (DE) synthesis (Zecca et al. 2004; Zucca et al. 2006). The source of the LC and SN MRI contrast has been suggested to relate to this high concentration of NM (Cassidy et al. 2019), based on the observation that the reduced MT effect is specifically observed in the LC and SN, the centers of NE and DA synthesis in the brain (Keren et al. 2009). Furthermore, histology studies showed a positive correlation between MT signal and the number of NM neurons or NM in the SN (Cassidy et al. 2019; Kitao et al. 2013), as well as spatial overlap between TSE hyperintensities and NM accumulation in the LC (Keren et al. 2015). The precise reason for this relationship between NM accumulation and reduced MT effects is, however, unclear. NM macromolecules are reported to be slightly paramagnetic and also tend to bind metals, which may shorten the T_1_ of the free water (Ju et al. 2013; Lee et al. 2016; Trujillo et al. 2016), thus potentially decreasing the longitudinal magnetization of both the free and bound water pools compared to adjacent brain matter (Fig. 1e). We should note though that the only NM model MRI study the authors are aware of, showed that NM on its own does not significantly affect MT behavior (Trujillo et al. 2016). Alternatively, it has been hypothesized that NM macromolecules may preferentially shorten the *T*_1_ of the bound pool (Langley et al. 2015), which would reduce the available magnetization for exchange (Fig. 1f). It is furthermore possible that the reduced MT saturation relates to a smaller macromolecular fraction, due to a lower concentration of lipids such as myelin on the cell’s axons, in line with the typical MT contrast observed between gray matter (GM) and white matter (WM). This may be further enhanced by the large cell size of noradrenergic neurons (up to 40 μm (Kimberly Simpson 2007)), which may result in a smaller bound-to-free water ratio (Fig. 1g). Finally, it was recently put forward that the large cell diameter of NA and DA neurons may produce a large intra-cellular free water proton pool which may show a short T_1_ due to a high concentration of paramagnetic ions, such as from Copper (Cu) within the cell body (Fig. 1h) (Watanabe et al. 2019). The last 2 hypotheses were further supported by a recent publication that showed smaller pool size ratio (Trujillo et al. 2019).

In this study, we aimed to disentangle the various sources of LC and SN MRI contrast. First, we applied a MT protocol at various frequency offsets and amplitudes along with *T*_1_ and *Τ*_2_* mapping in an NM phantom while manipulating the concentration of NM, to examine the effect of NM on MRI signal. Subsequently, we applied the same high-resolution protocol in vivo and compared our results with the phantom data, as well as with other adjacent regions of interest (ROIs). To confirm these results, we conducted an MT experiment with a short saturation pulse. The short MT experiment showed differential sensitivity to the macromolecular fraction compared to the *T*_1_ of the free and bound pool or the exchange rate between the pools, as we demonstrated through simulations. Finally, given the previously reported differences between young and older individuals in LC intensity, we also examined in vivo age-related differences in MT and *T*_1_, two contrast mechanisms that are intertwined in typical NM-MRI approaches.

## Methods

### Phantom construction

First, a NM phantom with varying NM concentrations was constructed. The purpose of the NM phantom was to examine possible *T*_1_/*T*_2_* and MT effects of NM on its own. The presence of NM macromolecules may shorten *T*_1_/*T*_2_ through a volume restriction mechanism due to their size or through a weak susceptibility effect (Bolding et al. 2013). Both of these mechanisms may facilitate a reduced MT. We should note that, within humans, NM forms complexes with metal ions, which may provide additional susceptibility contrast and reduced MT. Within the context of this study, we examined these susceptibility effects in vivo.

NM is rare in the human brain and hard to isolate in an amount sufficient for experiments. Therefore, the dopamine melanin cysteinyl-dopa, which is synthesized in the presence of L-Cysteine, (from now on referred to as DAM) melanin, is typically used as a substitute for NM (Trujillo et al. 2016). Molecules of the DAM model were, however, recently shown to have reduced hydrophilic behavior and different molecular interactions compared to brain tissue NM (Schroeder et al. 2015), which is likely to affect their MR behavior. Sepia melanin was suggested as a more accurate NM model due to its chemical composition, size, ease of isolation (from the Sepia Officinalis), and purity and surface characteristics (Schroeder et al. 2015). Both NM models were therefore employed in this study.

All chemicals were procured from Sigma Chemical Co. (St. Louis, MO, USA). The DAM melanin was synthesized using the description of Nguyen et al. (2002). Dopamine (H8502) and L-Cysteine (168,149) at a molar ratio of 6:1 were dissolved in 50 mM phosphate buffer (pH 7.2) and incubated at 37 °C with free access to air and vigorous stirring for 5 days. The resulting black pigment was collected after 10 min of centrifugation at 10,000 rpm, washed with 1% acetic acid and twice with distilled water. Finally, the melanin was dried under vacuum and kept at 4 °C until use.

**Fig. 2:**
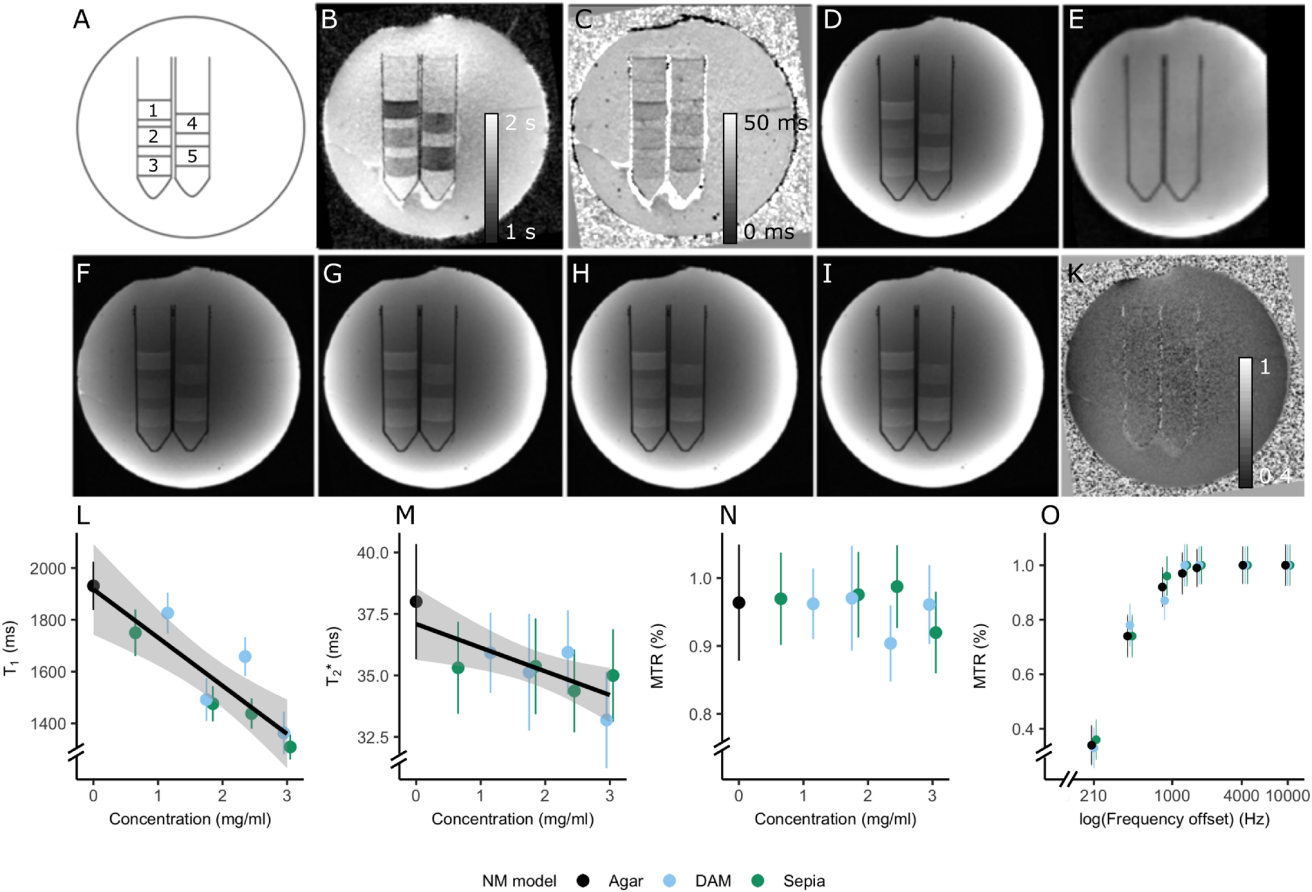
**a**–**k** Cysteinyl-dopa (DAM) and Sepia Neuromelanin (NM) models at different concentrations across modalities. **a** Scheme of each NM model’s placement: (1: Sepia, 2.4), (2: Sepia, 0.6) (3: DAM, 1.2) (4: DAM, 1.8), (5: Sepia, 1.8) (concentrations in mg/ml). **b***T*_1_ map. Note that shorter *T*_1_ is observed at higher concentrations for both models. **c** *T*_2_* map. *T*_2_* shortening is observed similar to the *T*_1_ case. **d** TFL (no MT applied). **e** B_1_ map, largely homogeneous. **f**–**i**: MT-TFL acquisitions, matched to the TFL (MT pulse amplitude = 6.75 μΤ). Frequency offsets are increasing from left to right [*f*: 430 Hz, *g*: 860 Hz, *h*: 1280 Hz, i: 1710 Hz]. Note that the MT-TFL scans appear similar to the TFL with no significant MT modulation. **k** MTR map for frequency offset = 860 Hz. No contrast can be seen between NM models, implying that no sizeable MT effects exist. **l**–**o**, MRI signal along NM models and concentrations ROIs (mean and SD plotted). **l** *T*_1_ values. A negative relationship between *T*_1_ and concentration was observed (*t*-stat =  – 4.172, *p* = 0.009). **m** *Τ*_2_* values. A trend for a significant relationship between *T*_1_ and NM models and concentrations was found (*f*(2,9) = 4.16, *p* = 0.079). The post hoc tests did not show a significant effect for either concentration or NM model on their own. **n** MTR values (MT pulse amplitude = 6.75 μΤ, frequency offset 860 Hz). **o** *z*-spectra for agar, DAM and Sepia (concentration = 1.8 mg/ml). Note that the rest of NM phantoms and a detailed report of the MT results are presented in S-Fig. 1

The NM phantom consisted of three 50 ml tubes containing several layers of agar (known to have similar relaxation properties to human brain tissue), doped with varying concentrations of Sepia and DAM melanin (0–3 mg/ml in steps of 0.6 mg; Fig. 2a). We should note that this concentration is higher than the natural range of NM concentration in LC (1000–3500 ng/mg wet tissue (Zucca et al. 2006)), since producing a NM phantom at naturally occurring concentrations would make the process prone to errors. Due to an error during the construction process, two concentrations, one of 0.6 mg/ml DAM and one of 1.2 mg/ml Sepia were not properly approximated and were not added in the final phantom.

To reduce variations in agar concentration, the base gel was made in a single batch by adding agar powder (product code: 05,040) to demineralized water, under heating, until a 4% (by weight) solution was achieved. The solution was cooled down to 45 °C, and the Sepia melanin (product code: M2649) and DAM were added. Each layer was left to cool down until it solidified before the next layer was poured. Between successive layers of melanin-agar, a layer of agar was poured to exclude the possibility of intermixing between layers. The tubes were placed within a plastic sphere (180 mm diameter) filled with agar to increase the loading of the RF coil. The agar for the surrounding sphere was made out of two batches due to limited production capacity and contained 0.71% NaCl to improve the RF conductivity and therefore reduce B_1_ inhomogeneities (NaCl was not added in the base gel used for the melanins to eliminate the possibility that NM would bind Na ions). The phantom was stored at 4 °C to prevent mold formation. Before MRI scanning, the phantom was allowed to reach room temperature to ensure *T*_1_ values comparable to the in vivo human brain.

### Data acquisition

Briefly, MR data from the NM phantom and 4 healthy volunteers (2 female, median age (range) = 23.5 (21–27) y.o.) were initially acquired in a 3 T Prisma scanner with a 64-channel head–neck coil (Siemens Healthineers, Erlangen, Germany) for an experiment focused on relaxometry and quantitative MT. To further unravel our results, a transient MT experiment was performed with the same scanner/coil combination on 5 additional participants (2 female, median age (range) = 25.6 (23–29) y.o.).

Finally, a separate dataset of 24 young (12 female, median age (range) = 22.5 (20–30) y.o.) and 15 older individuals (7 female, median age (range) = 71.1 (61–84) y.o.) was extracted from previous studies within our lab. These participants were screened to exclude a history of major psychiatric or neurological disorders, brain injury or brain surgery, taking medications that may influence cognitive functioning or not being eligible for MRI scanning. All participants had normal or corrected-to-normal visual acuity. Since depression has been linked to LC atrophy (Klimek et al. 1997), we included participants who scored within the normal range (range = 0–11; median = 2.5, IQR = 1.2–3.7) on the Hamilton Rating Scale for Depression (Hamilton 1960). The older individuals performed cognitively within the age-, education- and sex-adjusted norms on the Mini Mental State Exam (MMSE; median = 29; IQR = 28–30). These MRI data were obtained at a 7 T Magnetom Siemens with a 32-channel head coil (Nova Medical, Wilmington, MA, USA).

### Data acquisition for quantitative MT and relaxometry

A magnetization transfer-turbo flash (MT-TFL) sequence was acquired, consisting of a multi-shot 3D readout (TR = 538 ms, TE = 4.08, flip angle = 12°, voxel size = 0.4 × 0.4 × 1 mm^3^, number of slices = 30, partial Fourier = 6/8, TA = 3:30 min) with a center-out k-space sampling, preceded by a train of 20 off-resonant Gaussian sinc pulses (pulse-to-pulse time = 10 ms, individual pulse length = 5.12 ms, bandwidth = 250 Hz) (Priovoulos et al. 2018). The excitation was performed with a flip angle of 8° degrees to minimize direct saturation while retaining sufficient SNR. MT-TFL scans were done in blocks of 50, 100, 150 V MT pulse amplitudes (2.215, 4.430, 6.740 μΤ equivalent; continuous wave power equivalent (CWPE) for the whole MT train: 0.061, 0.107, 0.153 μΤ (Ramani et al. 2002); maximum amplitude chosen empirically to ensure SAR limit compliance within a given TR) with 7 frequency offsets (∆*f*) in each block (210, 430, 860, 1280, 1710, 4280, 10,000 Hz). The frequencies were chosen based on previous observations regarding maximum LC contrast (Priovoulos et al. 2018; Trujillo et al. 2015) and the order was randomized within each block. For the phantom, TFL scans with the MT pulses turned off were interleaved among each MT pulse amplitude acquisition block; these were used to control the MT signal for *T*_1_ changes due to temperature increase because of power deposition. The field-of-view (FOV) of the in vivo MT-TFL data was placed perpendicular to the brainstem, covering the area from the inferior border of the pons to the superior border of midbrain. The MT exchange is susceptible to *B*_0_ and *B*_1_ inhomogeneities, and therefore *B*_0_ (gradient echo Siemens fieldmap, voxel size = 3 × 3 ×  3 mm^3^, TE = 2.04/6.12 ms, flip angle = 60°, TR = 771.84 ms, TA = 1:43 min) and *B*_1_ (DREAM, voxel size = 3.5 mm^3^, TR/TE = 6 ms/3 ms, flip angle = 7°, TA = 3:30 min (Nehrke and Bornert 2012)) maps were recorded to correct for such effects.

To examine *Τ*_2_* and *T*_1_ effects of NM, a multi-echo gradient echo sequence (ME-GRE; TR = 31 ms, TE = 2.73/7.65/13.61/21.86 ms, flip angle = 12°, voxel size = 0.7 × 0.7 × 0.7 mm^3^, number of slices = 192, partial Fourier = 6/8, GRAPPA = 2, TA = 7:45 min) and a Magnetization Prepared 2 Rapid Acquisition Gradient Echo (MP2RAGE; TR = 5000 ms, TE = 2.98 ms, flip angle = 5°/4°, TI = 750/2570 ms, voxel size = 1 × 1 × 1 mm^3^, number of slices = 176, partial Fourier = 6/8, GRAPPA = 3, TA = 9:42 min) (Marques et al. 2010) were also acquired. MP2RAGEs were acquired both at the beginning and the end of the phantom session to further assess possible *T*_1_ increases due to heating of the phantom and scanner instabilities. B_1_ maps were also acquired at the beginning of each session. In total, the phantom scan lasted 3:30 h, while each participant’s scan lasted 2 h.

### Data analysis for quantitative MT and relaxometry

For the phantom (which due to the lack of circulation was expected to show increased heating), an approximately linear signal change was observed among the TFL acquisitions between blocks, presumably due to altered *T*_1_ because of heating. A linear regression was fitted to the TFL datasets (4 datasets acquired throughout the whole scan session). This heating effect was then regressed out of each MT dataset. Areas of interest (ROIs) were drawn within the layers of the varying-concentration NM models and in an adjacent reference agar region. The ROIs were inspected to ensure no voxels with air bubbles were present in the 2.73 ms echo image of the ME-GRE. A linear regression model with mean intensity as dependent and NM model and concentration as independent variable was fitted per scan.

**Fig. 3:**
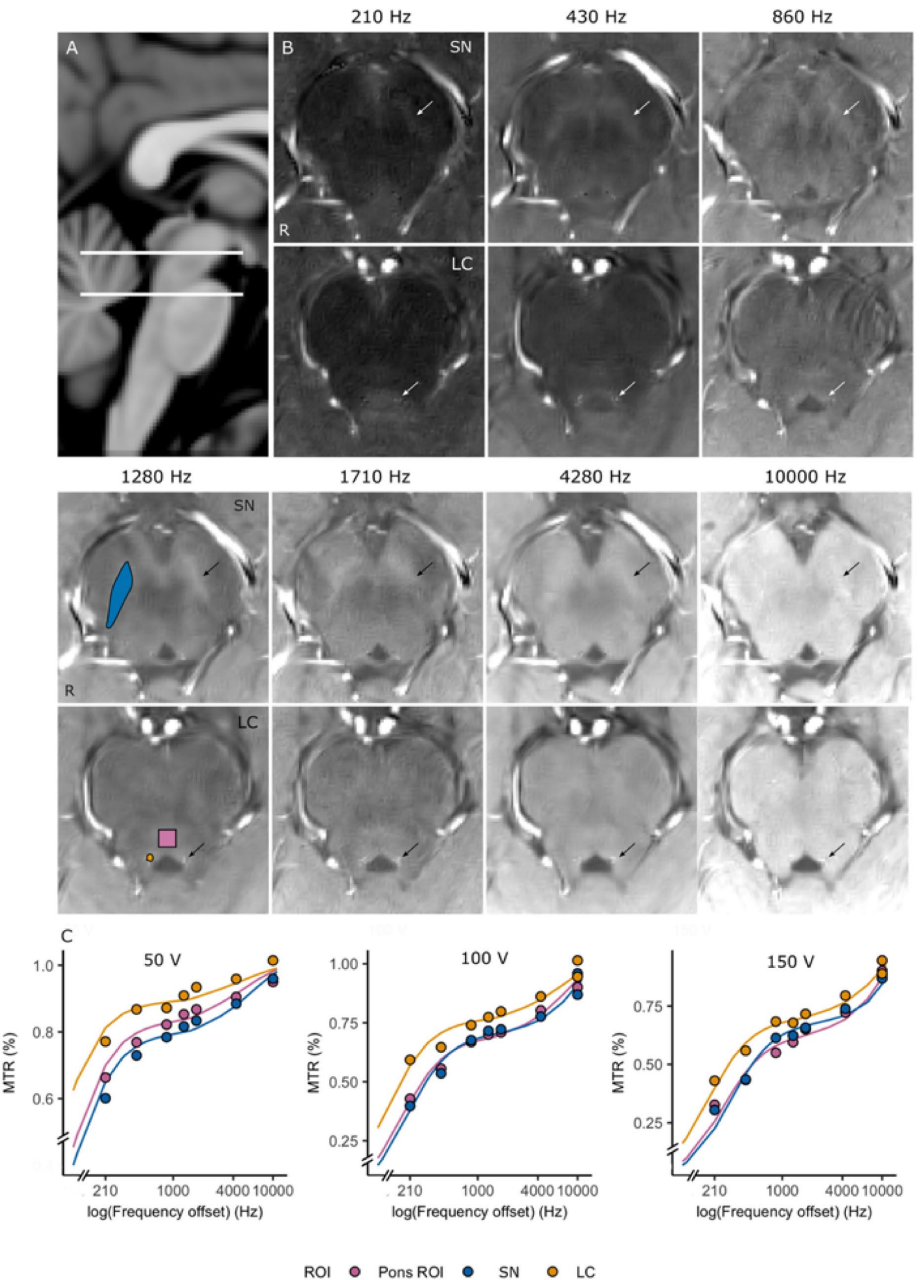
Locus Coeruleus (LC) and Substantia Nigra (SN) MT contrast along different frequency offsets. **a** Sagittal slice of the MNI template, indicating the levels of the axial slices shown (white lines). **b** Axial slices of SN and LC with the MT-TFL at 150 V pulse amplitude (*B*_1_ = 6.75 μΤ). The structures show as hyperintensities. The ROIs used to analyze the MT signal are shown for the right side for a frequency offset = 1280 Hz (*SN* – blue, *LC* – yellow, Pons reference gray matter *ROI* – pink; arrows). MT contrast between LC and SN and adjacent brain matter is visibly reduced for 210 Hz (i.e., in the direct saturation regime) and frequencies higher than 1710 Hz (i.e., outside of the resonance bandwidth of the bound pool). **c** z-spectra between the SN, the LC and the Pons ROI for different MT pulse amplitudes. The 2-pool model fits are overlaid as lines. Note that the LC shows reduced saturation compared to SN and Pons. This contrast is retained for higher frequency offsets, implying that it is not due to direct saturation

For each in vivo 3 T dataset, the middle TFL scan was registered to each MT-TFL scan with a 6-dof transform (Avants et al. 2011). To facilitate visualization and segmentations, the MT-TFL scans were denoised with a non-local means filter and assuming a Rician noise model with ANTs (Manjon et al. 2010). The LC and SN were identified as hyperintensities in the MT-TFL scan that was expected to show maximum MT contrast from previous research (150 V, 1280 Hz frequency offset (Jacobs et al. 2020; Priovoulos et al. 2018)) and ROIs were drawn (NP; Fig. 3b; median number of LC voxels = 84, interquartile range (IQR) = [68.2–98.5]; median number of SN voxels = 404, IQR = [358.3–472.5]). The ROI of the SN was drawn on the basis of the hyperintensity of the MT-TFL (i.e., ignoring the part of the SN that does not respond to NM-MRI but is more sensitive to *T*_2_*-weighted techniques (Langley et al. 2015)). Furthermore, a gray matter ROI (100 voxels) adjacent to the LC reference was drawn in the pontine tegmentum ventrally to the apex of the 4th ventricle at a plane 4 mm inferior to the superior LC boundary, as is typically done in LC imaging research (Castellanos et al. 2015; Clewett et al. 2016). All ROIs were projected on each MT-TFL scan with a rigid body transform.

MTR maps were calculated on a voxel-by-voxel basis, for both the phantom and in vivo data as: MTR $=\frac{\mathrm{TFL}-\mathrm{MT}}{\mathrm{TFL}} \times 100\%$. MT spectra were obtained using the normalized MT data as ${\mathrm{MT}}_{\mathrm{norm} }=1-\mathrm{MTR}$. *T*_1_ and *Τ*_2_* maps were fitted from the MP2RAGE and ME-GRE assuming single-exponential decays using a custom least squares algorithm implemented in MATLAB (Release 2015b, The MathWorks Inc., Natick, USA). *T*_1_ maps were corrected for *B*_1_ inhomogeneities using a *T*_1_–*B*_1_ dictionary (Marques and Gruetter 2013) in MATLAB. Average values for the MTR, *T*_1_, *Τ*_2_*, *B*_1_ and *B*_0_ maps were extracted for all ROIs, to use for the subsequent model fitting to ensure adequate SNR.

Subsequently, we attempted to quantify the MT exchange in the LC and surrounding regions in vivo to shed light onto their reduced MT effect. The MT exchange was assumed to be adequately described as the exchange between two pools (macromolecular (restricted, r) and free (f) water pool). In this case, the Bloch equations for the coupled system can be written as

1 $$\frac{{dM_{z}^{f} }}{{dt}} = R_{{1f}} \left( {M_{0}^{f}  - M_{z}^{f} } \right) - ~k_{{rf}} M_{0}^{r} M_{z}^{f}  + ~k_{{fr}} M_{0}^{f} M_{z}^{r}  + ~\omega _{1} M_{y}^{f}$$

2 $$\frac{{dM_{z}^{r} }}{{dt}} = R_{{1r}} \left( {M_{0}^{r}  - M_{z}^{r} } \right) - ~k_{{fr}} M_{0}^{f} M_{z}^{r}  + ~k_{{fr}} M_{0}^{r} M_{z}^{f}  + ~\omega _{1} M_{y}^{r}$$

3 $$\frac{{dM_{x}^{{f,~~r}} }}{{dt}} =  - \frac{{M_{x}^{{f,r}} }}{{T_{{2f,r}} }} + 2\pi \Delta f~M_{y}^{{f,r}}$$

4 $$\frac{{dM_{y}^{{f,~~r}} }}{{dt}}~~ =  - \frac{{M_{y}^{{f,r}} }}{{T_{{2f,r}} }} + 2\pi \Delta f~M_{x}^{{f,r}}  - \omega _{1} M_{z}^{{f,r}}$$

where*f* and *r* represent the free and bound pool, ${R}_{1r}$ and ${R}_{1f}$ are their respective longitudinal relaxation rates, *k*_rf/fr_is the exchange rate between the pools and $\Delta f$ is the frequency offset. It has been shown that if sufficient saturation is applied, then this system reaches a steady-state, where the above magnetization derivatives are 0 (Henkelman et al. 1993), in which case, the longitudinal magnetization of the free water pool can be expressed as

5 $$M_{z}^{f}  = \frac{{R_{{1r}} \left[ {\frac{{k_{{rf}} *f}}{{R_{{1f}} }}} \right] + W_{r}  + R_{{1r}}  + k_{{rf}} }}{{\left[ {\frac{{k_{{rf}} *f}}{{R_{{1f}} }}} \right]\left( {R_{{1r}}  + W_{r} } \right) + \left( {1 + \frac{{W_{f} }}{{R_{{1f}} }}} \right)\left( {W_{r}  + R_{{1r}}  + k_{{rf}} } \right)}}$$

where ${W}_{r}$ is the constant RF absorption rate due to the application of off-resonance saturation of amplitude ${\omega }_{CWPE}$ of the free pool given by ${W}_{f} ={\left(\frac{{\omega }_{1\mathrm{cwpe}}}{2\pi \Delta }\right)}^{2}\left(\frac{1}{{T}_{2f}}\right)$ and for the restricted pool ${W}_{r} = \pi {\omega }_{1\mathrm{cwpe}}^{2}G(\Delta , {T}_{2r})$. *f* is the pool size ratio between the bound and free pool (typically thought to express the concentration of lipids) defined by $f=\frac{{k}_{fr}}{{k}_{rf}}$ (Ramani et al. 2002).

Given the known relationship between the parameters, this model simplifies to 6 unknown terms (*f*, *k*_rf,_ *R*_1f_, *R*_1r_, *T*_2f_, *T*_2r_) that were fitted to the MT datasets. Since the R_1r_ is difficult to fit reliably, it was assumed to be equal to 1 s, similar to previous studies. The fitting of the two-pool MT model was performed with the package qMRILab (Cabana et al. 2015) in MATLAB assuming a super-Lorentzian lineshape for the *z*-spectra. The *B*_1_ and *B*_0_ estimates within ROIs were used to correct the nominal flip angles and frequency offsets. To estimate the pool size ratio *f* (thought to relate to the fraction of macromolecules, including lipids and therefore relating to myelin in the human brain), we expressed the longitudinal decay rate ${R}_{1f}$ as a function of the rest of the unknowns and the *B*_1_-corrected *T*_1_ values, extracted from the MP2RAGE (Ramani et al. 2002). For one representative participant, a white matter ROI was drawn in the crus cerebrus (Cassidy et al. 2019) and the z-spectra were plotted.

### Transient magnetization transfer experiment

Typical MT experiments, such as the one described above, apply intensive off-resonance saturation to approximate a steady-state of the longitudinal magnetization. This has the advantage of producing a large decrease of the signal and so increases sensitivity, while the assumption of $\Delta {M}_{\mathrm{free},\mathrm{ bound}}=0$ simplifies the signal description (Henkelman et al. 1993), thus facilitating “quantitative MT” approaches. While this has proven to be a useful approach for microstructural MRI quantification, the lengthy saturation renders the process more sensitive to the characteristics of the pulse, the *R*_2_ of the macromolecular pool (by comparison, following the application of a single pulse, the transversal magnetization relaxes within 1–2 ms) and the *R*_1f_ (due to the higher levels of direct saturation) (van Gelderen et al. 2017). Furthermore, the transient MT signal is differentially sensitive to the remaining physical parameters, such as *R*_1r_ and *f*. For the LC and SN MRI contrast in particular, where several parameters have been suggested as possible culprits, mapping the MT exchange timeline can shed light on the precise contrast mechanism.

To demonstrate the sensitivity of the signal to various mechanisms, first a set of simulations were conducted. Assuming a saturation pulse (or any RF pulse) and a 2-pool model, the longitudinal free water signal can be described by the Bloch equation including the coupled terms (Eq. 1). The analytical solution of Eq. 1 expresses the normalized longitudinal magnetization as a bi-exponential function:

6 $$M_{z}^{f}  = a_{1} e^{{ - \lambda _{1} t}}  + a_{2} e^{{ - \lambda _{2} t}} ~$$

with ${a}_{\mathrm{1,2}}$ and ${\lambda }_{\mathrm{1,2}}$ being functions of the relaxation and exchange rates and the initial magnetization of the free and bound pool. ${M}_{z}^{f}$ is expressed as fractional saturation ranging from 0 (fully relaxed) to 1 (fully saturated) to 2 (fully inverted). To simulate our signal, we calculated ${M}_{z}^{f}$ based on Eq. 6 while varying *R*_1f_ (0.25–2 s^−1^), *R*_1r_ (1–4 s^−1^; values chosen between the typically assumed *R*_1r_ and a recent estimate of it (van Gelderen et al. 2017)), *k*_rf_ (20–80 Hz (van Gelderen et al. 2017)) and *f* (1–18%, based on current estimates of macromolecular fraction, e.g., (Trujillo et al. 2017)). Since *k*_fr_ is expressed as a combination of *k*_rf_ and *f*, it was not simulated. The values of the remaining parameters at each simulation were fixed at ${R}_{1f}$= 1 s; ${R}_{1r}$= 1 s; *f* = 10%; *k*_rf_ = 15 Hz; ${M}_{z0}^{r}$= 1 (i.e., fully saturated); ${M}_{z0}^{f}$= 0.05 (to mimic partial direct saturation). Furthermore, the differential sensitivity of MT compared to an on-resonance inversion to variations in ${R}_{1f/r}$ was explored.

Following the simulations, data were acquired for 5 participants: a 2D MT-GRE acquisition was set up, consisting of a short saturation train of 2 pulses at 360 V (16.2 μΤ equivalent, 1200 Hz) followed by a simple GRE readout (0.5 × 0.5 × 2.5 mm^3^, TE = 5 ms, flip angle = 12°, TR = 531 ms, 2 slices, TA = 3:30 min). The delay between the MT and the readout was varied so that 9 points of the MT process were sampled (from the center of the saturation to the center of the readout, [12, 42, 87, 122, 162, 212, 262, 362, 512] ms). An extra delay was added after the readout so that the TR was consistent between acquisitions*.*

A MT-TFL scan like the one used for qMT was acquired for visualization purposes. ROIs, similar to the ones described above were drawn and projected onto each MT-delay dataset following the calculation of a 6-dof linear transformation (ANTs). Due to the reduced SNR and coverage of the 2D MT-GRE, the median across participants was estimated for each ROI and timepoint (individual data shown in Appendix).

The bi-exponential model of Eq. 6 was fitted to our data with iterative non-linear least square fitting in the R 3.5.1 package (custom script). While ${a}_{\mathrm{1,2}}$ and ${\lambda }_{\mathrm{1,2}}$ were estimated, we did not attempt to estimate the physical parameters derived from them (*R*_1r_, *R*_*1f*_, *k*_rf_, *f*) since this requires assumptions regarding the initial magnetization of each pool, resulting in 6 unknowns.

### Dataset comparing young and old subjects at 7 T

Finally, having examined the NM-MRI contrast mechanism in LC and SN in young people, we decided to examine its relevance in aging. NM-MRI signal differences have been reported several times between older and young participants and in relation to behavior (Betts et al. 2017; Clewett et al. 2016; Hämmerer et al. 2018; Liu et al. 2018), implying that the contrast mechanism is affected in aging. From other studies within our lab, we extracted a dataset of 24 young and 15 older individuals. For each individual, the dataset consisted of an MP2RAGE (TR = 5000 s, TE = 2.47 ms, flip angle = S 5°/3°, voxel size = 0.7 × 0.7 × 0.7 mm^3^, number of slices = 240) and an MT-TFL. The MT-TFL consisted of a multi-shot 3D readout (TR = 538 ms, TE = 4.08 ms, flip angle = 8°, voxel size = 0.4 × 0.4 × 0.5 mm^3^, FOV = 192 × 192 × 60, partial Fourier = 6/8) with center-out k-space sampling, preceded by 20 off-resonant Gaussian sinc pulses (pulse-to-pulse time = 10 ms, individual pulse length = 5.12 ms, bandwidth = 250 Hz, individual pulse B_1_ = 3.32 μT (260.88°), continuous wave power equivalent (CWPE) over the train duration = 0.084 μT (193.23°), offset frequency = 2000 Hz).

A T_1_ map was extracted from the MP2RAGE as described above (Marques et al. 2010). The MT-TFL and *T*_1_ map were registered to a study-specific template in the MNI 0.5 mm space using a diffeomorphic transform (ANTS 2.1). ROIs were drawn for the LC and SN by NP. The mean intensities per ROI were calculated. Since the MT-TFL scan is non-quantitative, a pons reference ROI was used for their normalization. A linear regression model with mean intensity as dependent and group as independent variable was fitted per ROI and scan. Given that motion affects the MRI signal and may differentially occur in each group, we calculated a retrospective motion metric (average edge strength; AES) with the homonymous Matlab toolbox (https://www.mathworks.com/matlabcentral/fileexchange/66002-average-egde-strength-aes) per scan. AES was entered in each regression as a confound variable.

## Results

### NM phantom

A NM phantom was successfully constructed, with the signal within the NM layers appearing largely homogeneous (Fig. 2e). The NM layers showed visibly shorter *Τ*_2_* and *T*_1_ values compared to agar. The MT-TFL showed contrast between the NM layers and agar albeit not specific to the MT, as can be seen from the similar signal in the *Τ*_2_*-weighted TFL image and the lack of contrast in the MTR image. For brevity, only one slice of the phantom at one amplitude is shown in Fig. 2. The results are all NM phantoms and amplitudes are shown in S-Fig. 1.

Multiple linear regressions were fitted for the *T*_1_, *Τ*_2_* and MTR (frequency offset = 860 Hz; amplitude = 150 V) maps. To examine the relationship between NM and *T*_1_, *Τ*_2_* and MT, the NM concentration and NM model were treated as independent variables and the signal as dependent variable. NM concentration and model were found to be significantly associated with the *T*_1_ estimate (F(2,9) = 10.9, *p* = 0.012, *R*^2^ = 0.788; Fig. 2b, l). Post hoc tests showed that higher NM concentrations were associated with lower *T*_1_ values (*t*-stat =  – 4.172, *p* = 0.009), but no significant difference existed between sepia vs agar (*t*-stat =  – 0.50, *p*-value = 0.636) or DAM vs agar (*t*-stat = 0.327, *p*-value = 0.756). A trend for a significant relationship between NM model and concentration and T_2_* values was also found (F(2,9) = 4.16, *p* = 0.079, *R*^2^= 0.542; Fig. 2c, m). Post hoc tests showed no significant effect for concentration (*t*-stat =  – 1.52, *p* = 0.189) or differences between sepia vs agar (*t*-stat =  – 1.455, *p*-value = 0.205) or DAM vs agar (*t*-stat =  – 1.317, *p* value = 0.244). The NM model and concentration did not significantly predict the MTR signal (*F* (2,9) = 0.476, *p* = 0.713, *R*^2 ^= 0.222). The regression was performed for the frequency offset in which maximum MT contrast between LC/SN and gray matter has been observed in vivo before (Priovoulos et al. 2018; Trujillo et al. 2015). Visual check confirmed a similar lack of contrast between NM models and agar across all frequency offsets (Fig. 2k, n, o).

Overall, these results suggest a potential *T*_1_ (and weaker *Τ*_2_*) modulation due to NM, though at concentrations that are unlikely to occur in in vivo tissue. No MT modulation due to NM was observed, implying that, even at this high NM concentration, the *T*_1_ shortening did not result in an observable MT decrease. Due to the lack of MT modulation with respect to different NM models, no further quantitative MT analysis was carried out.

### In vivo data

**Fig. 4:**
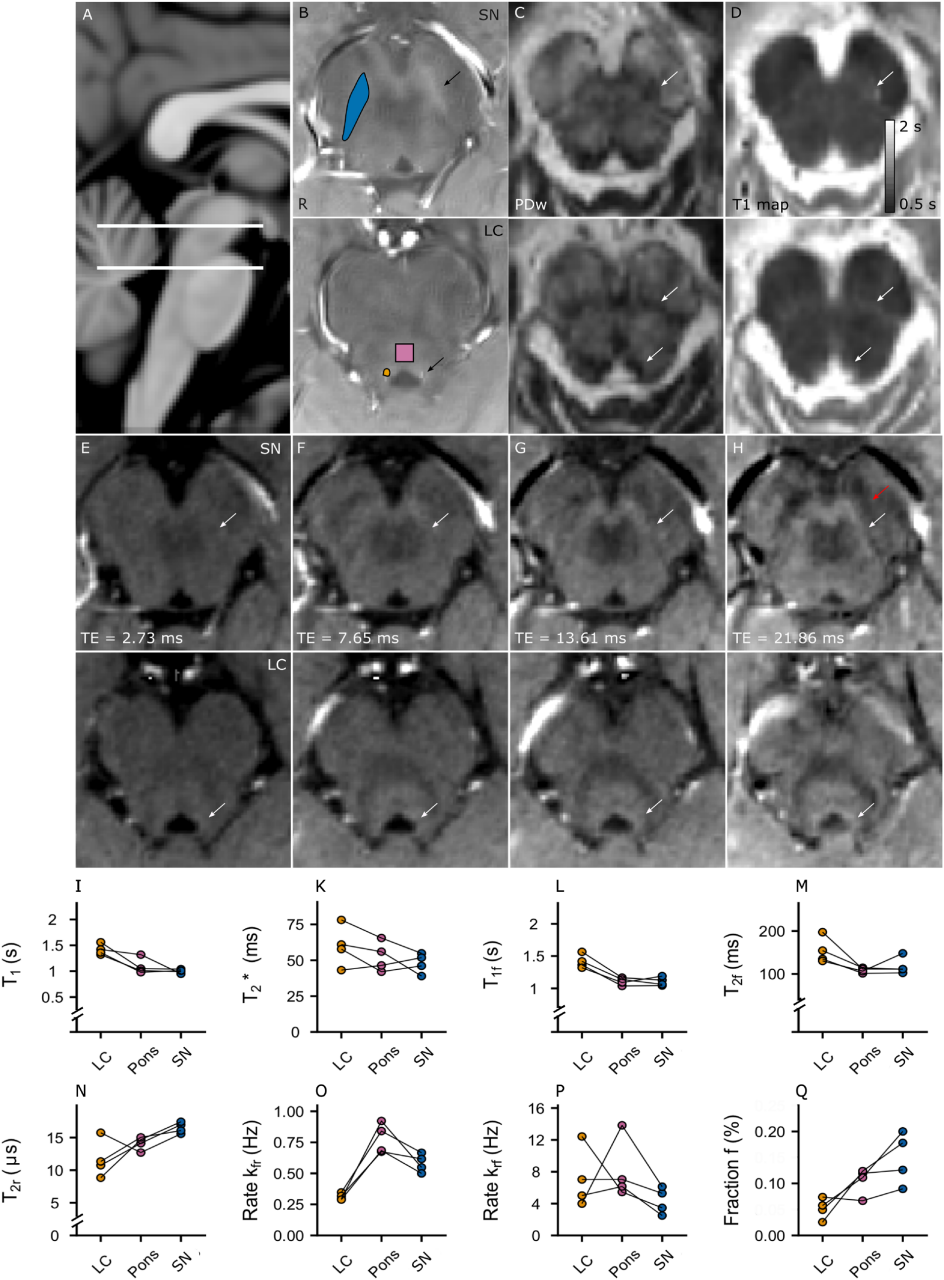
**a** Sagittal slice, indicating the axial slices shown in B-H at the level of the locus coeruleus (LC) and substantia nigra (SN) (white lines). **b** MT-TFL axial slices at the level of LC and SN (arrows). **c** MP2RAGE, PD-weighted image. Contrast compared to white matter is observed in the LC, the SN and the Pons ROI as expected from gray matter structures. **d** MP2RAGE, *T*_1_ map. Contrast associated with longer *T*_1_ values is observed in SN. **e–h**, GRE data across echo time, normalized to each echo’s mean signal intensity to facilitate contrast comparison. With longer echo times, the SN and the LC contrast compared to WM is becoming increasingly apparent, as expected due to the long *Τ*_2_* of free water associated with gray matter. **e** TE = 2.73 ms. No visible contrast can be seen in the areas of interest at the shortest echo (sensitive to the fast *Τ*_2_* decay of bound water protons). **f** TE = 7.65 ms. **g** TE = 13.61 ms. H, TE = 21.86 ms. Note the delineation between the SN and the iron-rich SN pars reticulata, which appears hypointense due to its short *Τ*_2_* (red arrow). **i** *T*_1_ values as fitted from an MP2RAGE. **k** *Τ*_2_* values as fitted from a 4-echo ME-GRE. Longer *T*_1_ and *Τ*_2_* are observed in the LC compared to the SN and the Pons ROI, typically associated with a higher proportion of free water. **l**–**q** Fitted values from a two-pool MT model. A longer *T*_1_ and *Τ*_2_ of the free pool, associated with a higher ratio of free water compared to macromolecular concentration, was fitted in LC. This may result in a reduction of the exchange rate from the free to the bound pool. The SN instead showed a higher fraction of lipids, compared to the LC and the Pons GM ROI

High resolution (0.4 × 0.4×1 mm^3^) data were collected for all participants with the MT-TFL across 3 MT pulse amplitudes and 7 frequency offsets (single scan duration 2 min 14 s). The frequency offset manipulation affected the contrast in the LC and SN (qualitatively one can observe that maximum contrast between them and the adjacent brain matter is evident in the range from 860 to 4280 Hz, i.e., within the frequency band where MT exchange is typically observed in brain tissue (Fig. 3), but reduces outside of this range). While both the LC and the SN showed similar z-spectra compared to the Pons GM ROI, the MT effect in LC was reduced, as expected. The *z*-spectra for the ROIs were approximated as a super-Lorentzian and the 2-pool model was fitted. From the fitting results in the 4 participants, the LC showed higher ${T}_{1}^{F}$(mean_LC_ = 1.42 s) and ${T}_{2}^{F}$(mean_LC_ = 0.15 s) of the free water and smaller *k*_fr_ (mean_LC_ = 0.31 Hz) compared to the Pons and SN, implying a lower macromolecular pool fraction *f* (mean_LC_ = 5.15%; Fig. 4l–q; Table 1). The SN compared to the Pons showed a higher macromolecular pool fraction *f* (mean_SN_ = 14.54% vs mean_Pons_ = 10.53%)*,* lower *k*_fr_ (mean_SN_ = 0.58 Hz vs mean_Pons_ = 0.78 Hz) and k_rf_ (mean_SN_ = 4.34 Hz vs mean_Pons_ = 8.12 Hz). The contrast ratios between the SN, the LC and the Pons are shown in S-Table 1. The z-spectra for all amplitudes for one representative participant including a white matter ROI (crus cerebrus) are shown in S-Fig. 2.

**Table 1:** Summary of the observed *T*_1_ and *Τ*_2_* values and the estimated parameters from the two-pool MT model per participant

ROI	Participant	*T*_1_ (s)	*T*_2_* (ms)	*T*_1f_ (s)	*T*_2f_ (ms)	*T*_2r_ (μs)	*k*_fr_ (Hz)	*k*_rf_ (Hz)	Fraction *f* (%)
LC	1	1.56	61.00	1.57	154.46	10.75	0.35	7.03	4.93
	2	1.32	78.13	1.36	134.75	15.74	0.29	4	7.37
	3	1.42	57.80	1.42	197.7	88.37	0.32	1.24	2.55
	4	1.36	43.13	1.32	130.3	11.34	0.29	5.01	5.76
SN	1	1.04	39.00	1.13	111.02	16.96	0.67	5.29	12.6
	2	0.99	54.86	1.04	102.51	15.57	0.59	6.12	8.96
	3	0.95	46.13	1.06	111.38	17.42	0.62	3.46	17.8
	4	1.01	51.84	1.19	148.07	16.07	0.50	2.5	20
Pons ROI	1	1.05	56.00	1.17	114.58	14.13	0.84	7.04	11.94
	2	1.01	65.53	1.04	101.5	12.69	0.92	1.38	6.67
	3	1.32	42.01	1.14	111.6	14.68	0.67	5.45	12.37
	4	0.98	46.42	1.09	106.56	15.03	0.68	6.15	11.12

*T*_1_ and *Τ*_2_* maps similarly showed higher estimates in the LC from the MP2RAGE and ME-GRE acquisitions (Fig. 4d, i, k). A baseline M_0_/Proton density weighted (PDw) image (that the MP2RAGE image is typically corrected for) can be conveniently extracted. The PDw image showed a high contrast with surrounding WM as would be expected in GM/WM regions (Fig. 4c). In the ME-GRE, a strong signal compared to surrounding WM, was visually observed at longer echo times in both the LC and partially in the SN, as would be expected in gray matter regions with large free water proton pools. This was in contrast to iron-rich adjacent regions, such as the SN pars reticulata (Fig. 4h). No visible contrast was observed in either the LC or the SN at short echoes (Fig. 4e). Following a single-exponential fit, an elongated *T*_2_* was detected in the LC (Fig. 4k), though no similar result was found for the SN, despite a visibly hyperintense signal at long echoes (Fig. 4h). It may be that a blooming artifact from the adjacent iron-rich part reticulata may have confounded our *T*_2_* estimate of the SN.

### Transient Magnetization transfer dataset

**Fig. 5:**
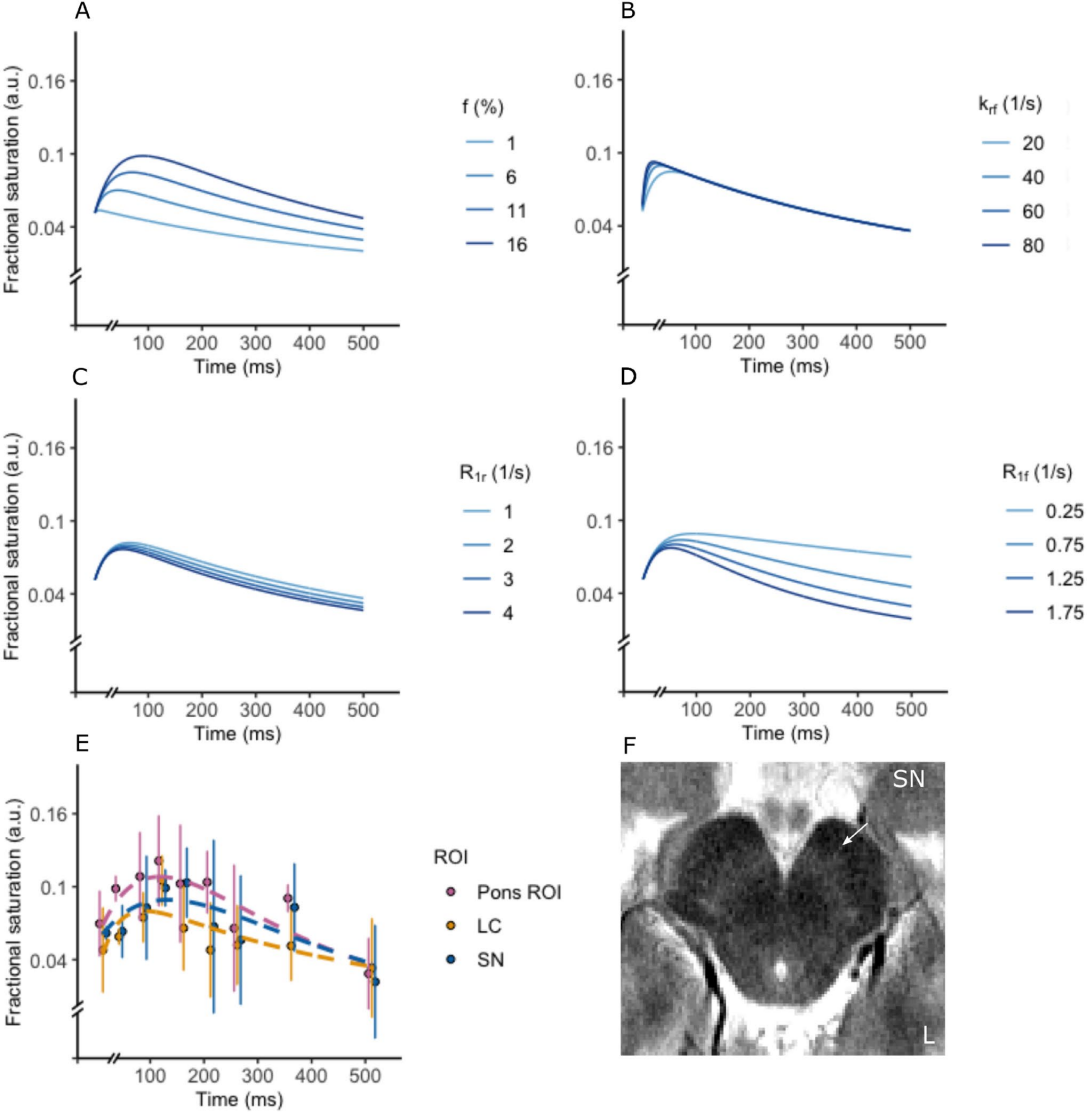
**a–d**, Simulations of the effect of exchange rate *k*_rf_, macromolecular fraction *f* and *R*_1f,r_ on the longitudinal free water signal (expressed as a fractional saturation) in a transient Magnetization Transfer (MT) experiment. Note that the effect of the exchange rate *k*_rf_ and *R*_1r_ is small (i.e., results in a reduced signal variation) compared to that of the macromolecular pool ratio *f* and *R*_1f_. Further note that *R*_1f_ variations result in signal differences more prominent at longer delay times, i.e., when the MT is reduced due to longitudinal recovery, thus resulting in a signal dominated by *R*_1f_ relaxation. Differences in *f* are instead more prominent at shorter delay times, which are dominated by the MT process itself. **e** Median and IQR (solid line) fractional saturation across participants between timepoints and ROIs (locus coeruleus (*LC*)—yellow; substantia nigra (*SN*)—blue; reference GM ROI in pons (*Pons ROI*)—Pink) in the transient MT experiment. The bi-exponential fits are shown as dashed lines. Note that maximum saturation is achieved approximately at 100 ms across ROIs. Reduced saturation is clearly observed for the LC and the SN along most of the timeseries, in accordance with reduced MT. **f** Sample slice of a representative transient MT acquisition at 122 ms, showing the SN (arrow). Note that SN can be localized as a hyperintensity with the transient MT approach, similar to the steady-state approach, despite the relatively small sensitivity of transient MT to variations in *k*_rf_ and *R*_1r_

To confirm the mechanism that results in MT contrast in the LC and SN, a transient MT experiment was conducted. As noted earlier, transient MT is less sensitive to *R*_2r_ variations, since transversal coherence of the restricted pool dissipates very quickly, similar to *R*_1f_ variations through direct saturation, as a single saturation pulse is applied. Furthermore, it can be shown that the transient MT timeline is differentially sensitive to k_rf_, *f* and *R*_1f,r_. To demonstrate this, simulations of the resulting fractional saturation of the longitudinal free water pool were conducted (Fig. 5a–d). It can be readily observed that the signal difference between a range of *R*_1r_ values is small (Fig. 5c). A similar argument can be made for variations in the exchange rate *k*_rf_ (Fig. 5b)*.* It is clear that larger signal variations are feasible as a function of *f* and *R*_1f_ (Fig. 5a, d). Signal variations due to *R*_1f_ are exacerbated though at longer delay times, while variation due to the macromolecular fraction *f* is exacerbated between 0 and 250 ms, i.e., in the MT-dominated regime.

In the transient MT acquisition, a hyperintensity in the SN could be clearly identified between 0 and 350 ms, similar to the steady-state approach (Fig. 5f), despite the reduced MT saturation. The median signal along timepoints was fitted with a bi-exponential model for each ROI (Fig. 5e; individual timeseries in S-Fig. 3). Maximum saturation was reached approximately at 100 ms, in accordance with literature (van Gelderen et al. 2017). Both the LC and the SN showed less signal suppression compared to the Pons GM ROI, indicating decreased MT. Overall, the fact that a hyperintensity can be clearly observed despite the reduced sensitivity of the method to *k*_rf_ and *R*_1r_ implies that the ROI signal differences are due to a difference in *f* or *R*_1f_. The fact that the signal difference can be observed in the initial part of the MT timeseries and dissipates in the R_1f_-dominated regime, implies sensitivity to *f*.

To further examine the likely signal differences due to variations in *R*_1f,r_, we repeated the simulation for on-resonance inversion (e.g., a *T*_1_-weighted acquisition) compared to off-resonance saturation (e.g., MT-GRE). From the simulation it became clear that, given *R*_1f,r_ variation, on-resonance inversion results in a much bigger contrast compared to off-resonance saturation (Appendix, S-Fig. 4), implying that on-resonance inversion methods, such as an MP-RAGE would result in larger contrast compared to an MT-GRE, given R_1f,r_ variation.

### Dataset comparing young and old participants at 7 T

**Fig. 6:**
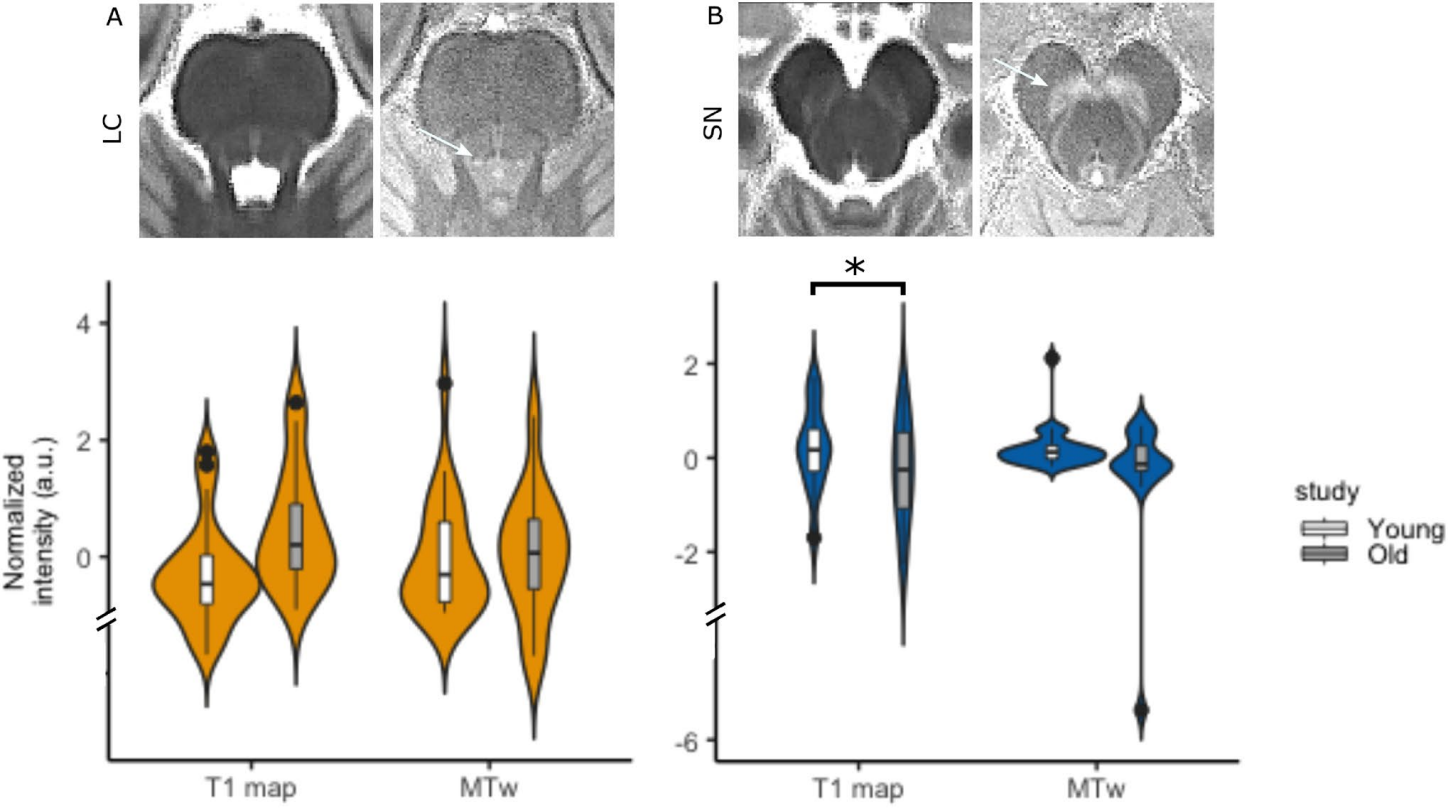
Age effect in LC and SN (white arrows) for *T*_1_ maps and MT-TFL. In the upper row, axial slices of the study-specific templates for each scan/map are shown. In the lower row, the *z*-scored intensity for the T_1_ map and the MT-weighted (MTw) image is shown. **a** LC. **b**, SN

Having examined the possible biological contributions to the NM-MRI contrast in young individuals, we examined whether the signal in the LC and SN is different between younger and older individuals (Table 2; Fig. 6). Both structures could clearly be identified in the MT-TFL study-specific template made with diffeomorphic transforms (Fig. 6a, b), implying that the registrations were of adequate quality to examine such small structures. Our results showed an elongation of *T*_1_ values for the LC in older compared to young individuals (Median_old_ = 1958 ms; IQR_old_ = 1904–2049 ms compared to Median_young_ = 1871 ms; IQR_young_ = 1826–1935 ms) though not for SN (Median_old_ = 1394 ms; IQR_old_ = 1351–1436 ms compared to Median_young_ = 1416 ms; IQR_young_ = 1392–1439 ms; data sampled at 7 T). No difference was observed in the MT-weighted scan (normalized with the Pons ROI) in older compared to younger individuals in both the LC (Median_old_ = 1.11 a.u.; IQR_old_ = 1.08–1.13 a.u. compared to Median_young_ = 1.09 a.u.; IQR_young_ = 1.07–1.13 a.u.) and the SN (Median_old_ = 1.01 a.u.; IQR_old_ = 0.98–1.25 a.u. compared to Median_young_ = 1.06 a.u.; IQR_young_ = 0.99–1.08 a.u.).

**Table 2:** Linear models per ROI and Scan with age group and average edge strength (AES) as independent and standardized intensity as dependent variable

ROI	Scan	Estimate	*t*-value	*p*-value	CI
LC	T_1_	1.467	3.086	0.015	0.504, 2.43
	MTw	0.783	0.706	0.646	– 1.47 to 3.04
SN	T_1_	– 0.158	– 0.305	0.762	– 1.211, 0.894
	MTw	– 0.925	– 0.866	0.646	– 3.09 to 1.24

Estimates indicate the average difference in standardized intensity for the old group compared to the young group

*P*-values were adjusted for multiple comparisons using the False Discovery rate

## Discussion

The LC undergoes structural and functional changes during aging and several neurodegenerative diseases that relate to behavioral deficits (Hämmerer et al. 2018; Jacobs et al. 2015, 2018; Liu et al. 2018; Sasaki et al. 2006; Trujillo et al. 2017). MT and TSE signal in the LC and the SN, the so-called NM-MRI, has been related to the presence of NE and DA neurons or NM macromolecules (Cassidy et al. 2019; Keren et al. 2015; Kitao et al. 2013), while signal differences in these regions are commonly observed in aging and neurodegenerative diseases compared to young people (Clewett et al. 2016; Hämmerer et al. 2018; Liu et al. 2018; Takahashi et al. 2015). The MRI signal in these regions may therefore have predictive value for the onset of diseases such as Alzheimer’s or Parkinson’s disease, but the precise contrast mechanism is unclear, with several hypotheses relating to short *T*_1_ of intra-cellular, free water or bound pool or susceptibility effects being put forward (Sasaki et al. 2006; Trujillo et al. 2016; Watanabe et al. 2019). In this study, we performed a series of experiments including quantitative and transient MT, *T*_2_* and *T*_1_ approaches on a phantom and in vivo to examine the likely contributing biological correlates. In our phantom, we were not able to detect a relationship between NM concentration on its own and MT. Furthermore, based on the transient and steady-state MT and the *T*_1_ and *T*_2_* decays, we did not detect evidence of a *T*_1_ or *T*_2_ shortening for either the free or bound water pools in the LC and SN. Based on the 2-pool fits and the transient MT experiment that is differentially sensitive to the physical parameters involved, we conclude that a reduced MT in the LC seems to be facilitated by a lower macromolecular pool size ratio. Age-related differences were detected in the *T*_1_ measurements in the LC, suggesting that the above mechanisms may be relevant in clinical research.

The LC and SN are unusual in that the macromolecule NM accumulates in organelles in its neurons (Zucca et al. 2006). These sites are also sensitive to implicit (TSE) and explicit (MT-based) off-resonance saturation, suggesting a larger bandwidth around the Larmor frequency due to differences in the macromolecular content. As such, the MR contrast in the SN and the LC have been suggested to relate to their high NM concentration (Cassidy et al. 2019; Sasaki et al. 2006). To examine if we could detect such a link, we created a phantom including two NM models (DAM and Sepia) at varying concentrations, adding to the only other study of NM-MRI properties that was performed with the DAM model (Trujillo et al. 2016). NM showed a *T*_1_ and a potential *T*_2_* shortening with increasing concentration (Fig. 2). This suggests that NM, a large macromolecule that tends to accumulate in granules (Zecca et al. 2002), may speed up the spin–lattice relaxation, potentially due to volume restriction. No difference in *T*_1_ or *T*_2_* was found between the NM models, implying similar MR properties. We should note that our phantom employed NM concentrations substantially higher than normally encountered in the human brain (Zecca et al. 2004). In vivo, no *T*_1_ or *T*_2_* shortening were observed in the LC in this (Fig. 4) or in previous studies (Langley et al. 2015; Priovoulos et al. 2018). Furthermore, and despite the high NM concentration, the *T*_1_ shortening we observed was not strong enough to result in a measurable MT decrease (Fig. 2). We therefore did not manage to establish a link between NM concentration on its own, *T*_1_ shortening and MT decrease. The lack of an MT effect from either of the NM models further suggests that NM itself did not directly exchange magnetization with free water protons, in line with the fact that NM shows reduced solubility in water (Zecca et al. 2001), so it does not bind water protons.

Given the small size of the LC (German et al. 1988) and the intense saturation needed, the in vivo experiment is particularly challenging. Our efficient MT-weighted sequence made it possible to sample the MT contrast in the LC and SN at high resolution across multiple combinations of off-resonance amplitude and frequency offsets within a time frame feasible in a single scan session. The decreased MT contrast close to resonance implies that this is a true MT effect and not due to direct saturation. The *Z*-spectra were modeled as a super-Lorentzian and a two-pool MT model was fitted to the data. The fitted parameters showed a decreased macromolecular fraction *f* in the LC and a longer *T*_1_ and *T*_2_ of the free pool compared to the Pons ROI. These results are in agreement with a recent qMT study from Trujillo and colleagues that, using a single-point fit in a clinically relevant acquisition time showed a lower macromolecular-to-free water fraction in the LC (Trujillo et al. 2019).

Alternative explanations of the reduced MT effect in the LC have been suggested, including the presence of copper ions in the intra-cellular water, i.e., a fast-decaying longitudinal magnetization of the free pool (Watanabe et al. 2019) or a fast-decaying longitudinal magnetization of the bound pool, potentially due to paramagnetic ions. In this study, we were not able to detect a *T*_1_ decrease in the MP2RAGE-derived T_1_ maps in the LC in this or our previous study (Priovoulos et al. 2018). Furthermore, the qMT steady-state fits in the LC imply an elongated rather than a decreased *T*_1f_ and *T*_2f_. Finally, as we showed in our 2-pool simulation, a decreased *T*_1f_ would be more readily detected by an on-resonance inversion than a MT experiment, which does not seem to be the case experimentally. To examine whether a rapid longitudinal decay was responsible for the LC and SN MR behavior, we performed a transient MT experiment, where the timeline of the saturation due to MT was sampled. Our simulations showed such an experiment would show reduced sensitivity to *T*_1r_ or the exchange rate *k.* Nonetheless, the MT contrast of the SN and LC was retained, while the median MT timeseries of the LC matched well with our simulations of a decreased *f*. Finally, and similarly to T_1f_, our simulations show that a decreased *T*_1r_ would be more readily detected by an on-resonance inversion than a MT experiment. Our results therefore imply that in a healthy young sample, the less-reduced signal after off-resonance saturation in the LC relates to a lower fraction *f* (i.e., more free water and/or less macromolecules).

As described in the literature (Kimberly Simpson 2007; Watanabe et al. 2019), NE and DA neurons may reach an unusually large cell diameter which may in turn result in a large intra-cellular water pool. This could reduce the macromolecular-to-free water protons fraction *f* and result in a brighter signal following off-resonance saturation of the macromolecular pool.

Intra-cellular water tends to have longer *T*_1_ and *T*_2_ compared to the extra-cellular water likely due to higher freedom of movement (Sati et al. 2013), which may further explain the *T*_1_ elongation detected in the LC.

In NM-MRI research, positive correlations have been reported between various MRI contrasts and the number of NM-accumulating NE and DA neurons (TSE (Keren et al. 2015); MT-GRE (Kitao et al. 2013); spin echo (Lee et al. 2020)). If a tissue consisting of large NE neurons is characterized by a lower fraction *f*, this may indeed render MR contrast an indirect measure of NM-rich cell density in healthy young individuals. Such a relationship may exist across different MR techniques, since water and macromolecular concentration are two of the main contributors in most ^1^H MRI contrasts, including *T*_1_ and *T*_2_ (Stüber et al. 2014). Indeed, recently a *T*_1_-weighted approach was put forward to image the LC (Betts et al. 2017), though MT may further enhance such contrast by increasing sensitivity to *f*. Such a link between MRI signal and NE neurons may also explain the reported correlations between NM-MRI signal intensity and behavior-relevant features, such as cognitive reserve or emotional memory (Clewett et al. 2016; Hämmerer et al. 2018).

Increased suppression compared to the LC was detected in the SN in both steady-state and transient MT experiments (Figs. 3, 5), despite that the same contrast mechanism is typically assumed for both. This implies a higher pool fraction size in SN, a result in agreement with the other recent qMT study on LC and SN based on single-point fits (Trujillo et al. 2019). We should note that differences in the accumulation of NM and toxic metals bound to NM have been reported to exist between the SN and the LC (Zucca et al. 2006), which may relate to the increased MT effect in the SN. It has been demonstrated that the organelles that contain NM also tend to accumulate undegraded proteins and lipid bodies (Fedorow et al. 2006; Zecca et al. 2008; Zucca et al. 2018) that can be surprisingly big (up to 1 μm) compared to the NE and DA cell size. One can tentatively speculate that the lipid bodies bound to organelles that also enclose NM granules can get saturated by off-resonance pulses and themselves induce signal suppression. The presence of iron can further complicate the examination of MT in the SN (van Gelderen et al. 2017).

In the LC and the SN literature, several studies have reported NM-MRI signal differences in relation to age-groups or neurodegenerative conditions (Clewett et al. 2016; Hämmerer et al. 2018; Takahashi et al. 2015; Xing et al. 2018). In our own comparison between young and older subjects, we found that aging was associated with a longer *T*_1_ in the LC. Longer *T*_1_ in subcortical regions during aging is a common finding and may relate to volume loss, e.g., due to the reduced density of the long projections of the LC (Keuken et al. 2017; Okubo et al. 2017; Rorabaugh et al. 2017; Steen et al. 1995). Interestingly, no such effect was detected in the SN (Fig. 6). No significant difference was detected in the MT-weighted scans, following normalization with a reference ROI and a regression of a motion estimate during scanning. Non-quantitative scans, such as most typical implementations of NM-MRI (TSE, MT-TFL, T_1_w-TFL) combine contributions from several possible contrast mechanisms (PD, *T*_1_, *T*_2_, MT). Furthermore, reference ROIs can have their contributions from age-related changes or subclinical pathology that may confound results. In future studies, quantitative methods which isolate the contrast in question, as done for example in MTR or MP2RAGE (Marques et al. 2010) may help to increase sensitivity.

There are several limitations to this study. First, several types of NM exist (Engelen et al. 2012; Fedorow et al. 2005). While we tried to examine two models of them, it is not clear how these relate to the NM in the LC or the SN. Both our models though showed similar relaxation and MT properties, while brain NM macromolecules largely do not bind water protons (Engelen et al. 2012) so it unlikely that NM participates directly in MT. Furthermore, NM in humans typically binds with metals and the subsequent susceptibility effect reduced MT in a phantom (Trujillo et al. 2016). In this study, no metal ions were added during the phantom construction, but we examined the effects of iron in vivo. We should note, however, that, while iron tends to accumulate in the SN, this is much lower in the LC (Zucca et al. 2006), a result supported by susceptibility-weighted MRI (Priovoulos et al. 2018). Our results should be replicated in phantoms using NM extracted from ex vivo tissue (Cassidy et al. 2019), which is challenging given the low NM concentration in humans. Second, while the SN NM-MRI ROI showed little susceptibility contrast, there is high concentration of paramagnetic ions in the adjacent part of the SN (that is sensitive to T_2_* techniques) (Langley et al. 2015). The presence of those may have partially confounded our qMT model estimate, by reducing the transversal coherence. Third, while our results show that decreased MT can be produced in the LC and the SN under conditions that are predominantly sensitive to the macromolecular fraction (transient MT), the possible contrast sources are not necessarily mutually exclusive: for example, a lower macromolecular fraction can facilitate a slower longitudinal relaxation, even when accounting for MT effects, through thermal relaxation. As noted earlier, water and lipid concentration underlie most ^1^H MR contrasts. Finally, we should caution that in the young vs old comparison, no acquisitions without the off-resonance pulse were available to create MTR maps that would reduce scanner and sequence-specific effects. These results should be replicated with quantitative metrics.

The NM-MRI is getting increasing attention in cognitive and clinical neuroscience. We employed simulations, *T*_1_ and *T*_2_* mapping, quantitative and transient MT to probe the tissue properties of the LC and the SN both with a dedicated phantom and in vivo in order to disentangle the contrast’s mechanisms. We conclude that the LC tissue contrast in NM-MRI in healthy young individuals is likely related to a lower macromolecular pool fraction *f*, a feature potentially specific to the NE neurons’ physiology. We further show that an increase in *T*_1_ may occur in older compared to young individuals in the LC. We speculate that the longer *T*_1_ may reflect neuronal shrinkage or reduced density of the long projections of the LC due to aging or disease-related processes.

## Electronic supplementary material

Below is the link to the electronic supplementary material.

Supplementary file1 (DOCX 1305 kb)

## Data Availability

The custom code is available from the authors upon request.
