# Supplementary material for: Unraveling the contributions to the neuromelanin-MRI contrast
Source: Brain Struct Funct. 2020 Oct 22;225(9):2757–74. doi: 10.1007/s00429-020-02153-z (PMC7674382; doi:10.1007/s00429-020-02153-z)
Supplement: Supplementary file 1 — Supplementary file1 (DOCX 1305 kb) [file 429_2020_2153_MOESM1_ESM.docx]

Appendix

S1 – Magnetization transfer in the Neuromelanin phantoms


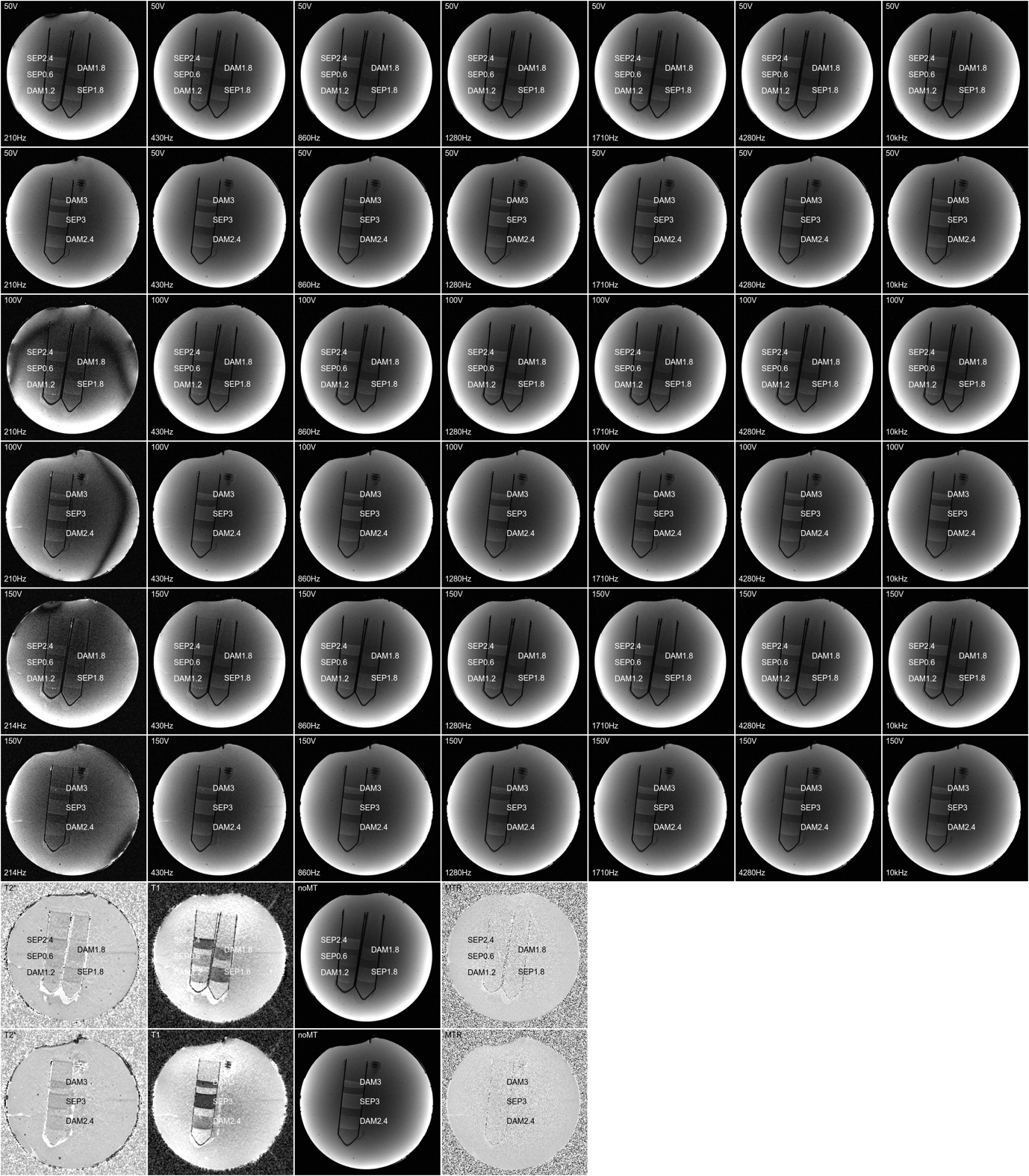


S-Figure 1: Neuromelanin phantom results for all models and concentrations. 2 axial slices are shown at even and odd rows so that all phantoms can be seen). First 6 rows, MT-TFL acquisitions at different amplitudes (rows) and frequencies (columns). Bottom 2 rows, T_2_*, T_1_, TFL and MTR map (150V, 860Hz). Each image is annotated with the phantoms it includes. DAM = Cysteinyl-dopa. SEP = Sepia Neuromelanin. The numbers show the concentration in mg/ml. Note that there is no obvious modulation by different frequencies and amplitudes of saturation.

S2 – steady-state Magnetization Transfer in vivo

| Saturation amplitude (V) | Frequency offset (Hz) | (LC-Pons)/Pons | (SN-Pons)/Pons |
| --- | --- | --- | --- |
| 50 | 210 | 0.316 | -0.068 |
|  | 430 | 0.289 | 0.006 |
|  | 860 | 0.243 | 0.115 |
|  | 1280 | 0.140 | 0.048 |
|  | 1710 | 0.102 | 0.011 |
|  | 4280 | 0.100 | 0.022 |
|  | 10000 | 0.049 | -0.035 |
| 100 | 210 | 0.385 | -0.072 |
|  | 430 | 0.164 | -0.035 |
|  | 860 | 0.108 | 0.013 |
|  | 1280 | 0.105 | 0.022 |
|  | 1710 | 0.125 | 0.018 |
|  | 4280 | 0.073 | -0.033 |
|  | 10000 | 0.068 | 0.010 |
| 150 | 210 | 0.163 | -0.093 |
|  | 430 | 0.129 | -0.051 |
|  | 860 | 0.061 | -0.046 |
|  | 1280 | 0.066 | -0.043 |
|  | 1710 | 0.077 | -0.039 |
|  | 4280 | 0.060 | -0.022 |
|  | 10000 | 0.068 | 0.010 |

S-Table 1: Contrast ratio for the LC-Pons and the SN-Pons.


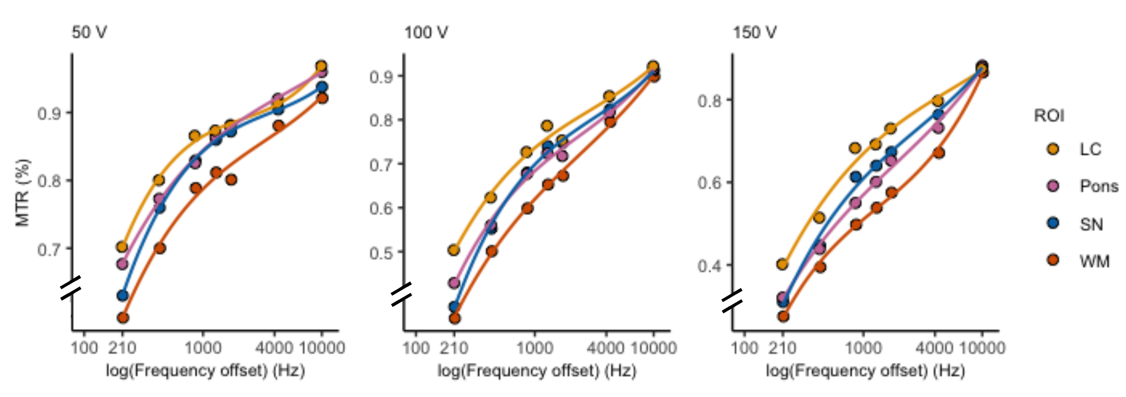


S-Figure 2: z-spectra between the SN, the LC, the Pons gray matter ROI and a white matter ROI (crus cerebrus; WM) for different MT pulse amplitudes (50-left to 150 V-right) for a representative participant.

S3 – transient Magnetization Transfer

Individual transient Magnetization Transfer timeseries


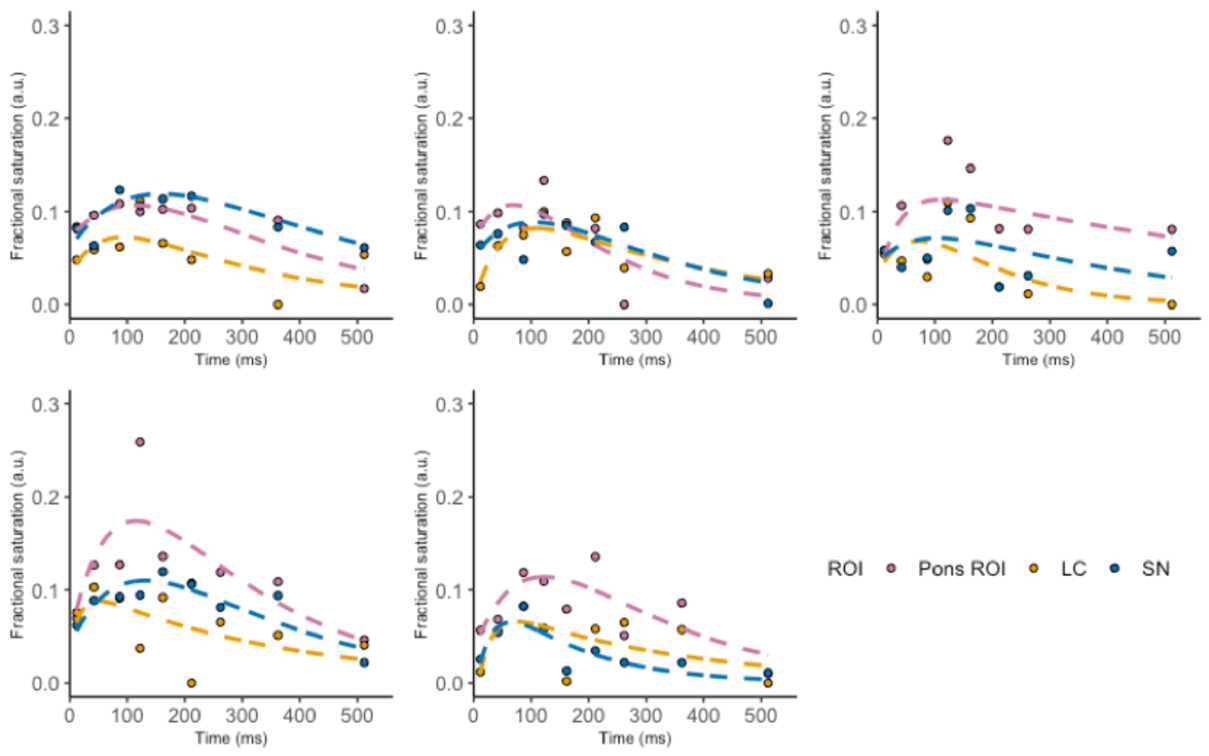


S-Figure 3: Individual transient Magnetization Transfer timeseries (locus coeruleus (LC) - yellow; substantia nigra (SN) - blue; reference GM ROI in pons (Pons ROI) - Pink). The individual bi-exponential fits are shown as dashed lines.

Simulation of inversion compared to magnetization transfer

Here we compared the normalized longitudinal saturation’s sensitivity to variations in R_1f,r_ following the application of an on-resonance inversion pulse compared to an off-resonance saturation pulse. We simulated the signal in both MT and inversion as an identical 2-pool model, based on the longitudinal free water Bloch equation with coupled terms (eq. 8 of the main text). The fractional saturation then ranges between 0 (no saturation) to 1 (fully saturated) to 2 (fully inverted). We assumed that the off-resonance pulse fully saturated the macromolecular pool (i.e., fractional saturation at 0 ms = 1) but did not perturb at all the bound pool (i.e., fractional saturation at 0 ms = 0). We further assumed that the inversion pulse fully inverted the free water pool (i.e., fractional saturation at 0 ms = 2) but did not perturb at all the bound pool (i.e., fractional saturation at 0 ms = 0). We further assumed that k_rf_= 15 Hz, R_1r_= 1 s^-1^ and f = 10%. Based on that, the signal evolution was plotted (S-Figure 2). Note that the on-resonance inversion results in much bigger signal difference with varying R_1f,r_. This implies that if a difference in R_1f_ or R_1r_ existed in the LC or SN, it would be more readily detected with an inversion experiment, e.g., an MP-RAGE, rather than an off-resonance magnetization transfer experiment.


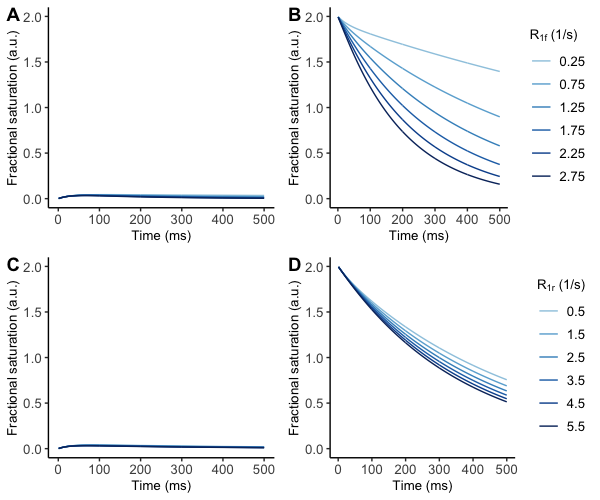


S-Figure 4: Simulation of normalized longitudinal magnetization at various R_1f_ (A-B) for off-resonance saturation (A) and for on-resonance inversion (B). Simulation of normalized longitudinal magnetization at various R_1r_ (C-D). Note that on-resonance inversion results in bigger signal variation (i.e., increased contrast) as a function of R_1r,f_ relaxation rates, compared to magnetization transfer.
